# Academic burnout in higher education: a multimodal study of resting-state EEG microstate correlates beyond trait anxiety, depressive symptoms, and self-efficacy in Chinese undergraduates

**DOI:** 10.3389/fpsyt.2026.1867360

**Published:** 2026-07-15

**Authors:** Jianxu Wang, Meng Zhang, Liguo Zhang

**Affiliations:** 1 Faculty of Education, Shaanxi Normal University, Xi’an, Shaanxi, China; 2 College of Education Science, Xinyang Normal University, Xinyang, Henan, China; 3 Department of Psychology, Henan Medical University, Xinxiang, Henan, China

**Keywords:** academic burnout, academic stress, depressive symptoms, EEG microstates, higher education, resting-state EEG, self-efficacy, trait anxiety

## Abstract

**Background:**

Academic burnout is a mental-health-relevant outcome of higher-education stress, but its relationship with spontaneous brain dynamics remains unclear. EEG microstates are quasi-stable scalp-potential configurations indexing millisecond-level transitions between large-scale resting brain states. This study tested whether resting-state EEG microstate dynamics are associated with academic burnout beyond trait anxiety, depressive symptoms, and general self-efficacy as perceived coping capacity.

**Methods:**

Participants were 330 Chinese undergraduates (94 men, 236 women; mean age = 18.31 years, SD = 0.84). They completed self-report measures and an eyes-closed resting-state EEG recording. Academic burnout total score was the primary outcome. Covariate-adjusted partial correlations examined burnout–microstate associations. Hierarchical regression tested the incremental value of selected microstate markers.

**Results:**

Higher academic burnout was associated with higher trait anxiety (r = 0.539, p < 0.001), higher depressive symptoms (r = 0.526, p < 0.001), and lower general self-efficacy (r = -0.474, p < 0.001). After adjustment for age, sex, trait anxiety, depressive symptoms, and general self-efficacy, greater burnout was associated with shorter microstate D duration (partial r = -0.196, q = 0.0049), higher microstate C occurrence (partial r = 0.172, q = 0.0081), shorter mean microstate duration, and higher mean occurrence. The psychological block explained substantial variance in burnout (R² = 0.417); adding microstate D duration and microstate C occurrence produced a small but significant improvement in model fit (ΔR² = 0.025, p = 0.001). In a scale-midpoint sensitivity analysis, the higher-burnout group showed shorter class D duration after covariate adjustment (adjusted B = -3.38 ms, 95% CI [-6.12, -0.64], p = 0.016). Dimension-specific analyses indicated that microstate associations were mainly evident for dejection and low sense of accomplishment, not the behavioral (improper-behavior) component.

**Conclusion:**

Academic burnout in undergraduates was primarily linked to internalizing distress and lower perceived coping capacity. Resting-state EEG microstate dynamics, especially shorter class D duration, provided modest incremental information, suggesting reduced temporal persistence of a canonical resting-state configuration among students with higher burnout. These findings do not establish a diagnostic EEG marker or a burnout-specific neural mechanism, but support multimodal, context-sensitive research on academic stress and mental health in higher education.

## 1 Introduction

Academic stress is one of the most prevalent and consequential forms of stress in higher education and is increasingly linked to poorer mental health, emotional dysregulation, and impaired academic functioning (1–3). Contemporary work suggests that student distress reflects not only workload, but also transitional demands, perceived performance pressure, financial and interpersonal strain, and uneven access to support within higher-education systems (4–6). This issue is especially salient in adolescents and young adults, whose university years overlap with the peak period of onset for many internalizing difficulties (7, 8). Against this background, higher education has become an increasingly important setting for research that integrates academic stress, mental health, behavior, and psychobiological markers in university populations.

Academic burnout is especially important within this broader framework because it captures the cumulative emotional and motivational costs of sustained study-related strain. In university students, burnout is typically conceptualized as a multidimensional syndrome comprising emotional exhaustion or dejection, disengagement from study, and diminished academic efficacy or accomplishment (9–11). Higher burnout has been linked to poorer well-being, reduced engagement, and worse academic functioning (12, 13). At the same time, burnout should not simply be collapsed into depression. Recent network evidence in college students indicates that burnout and depression are closely related but still distinguishable constructs, with burnout retaining its multidimensional structure (14).

Trait anxiety, depressive symptoms, and general self-efficacy were included as psychological reference variables rather than as novel determinants of burnout. Trait anxiety indexes relatively stable threat sensitivity, depressive symptoms index current affective burden, and general self-efficacy was used here as an operational index of perceived coping capacity, that is, a student’s generalized belief that he or she can manage demands and stressors (15–17). Recent work in Chinese undergraduate samples suggests that academic burnout is closely related to anxiety and depressive symptoms, whereas academic self-efficacy functions as a meaningful protective factor (18–20). More broadly, self-efficacy has also been linked to lower depressive symptomatology in Chinese college students (21). These variables therefore provide an appropriate psychological reference framework for testing whether academic burnout is associated with psychobiological variation beyond established self-report correlates. In the present study, trait anxiety was not treated as a new explanatory claim. Rather, it was included as a stringent covariate and psychological benchmark, so that any EEG microstate association could be interpreted as information beyond a well-established internalizing vulnerability factor.

Despite this progress, most studies of academic burnout remain based exclusively on self-report. As a result, it is still unclear whether burnout severity is accompanied by measurable variation in spontaneous brain dynamics beyond what can already be explained by established psychological correlates. Resting-state EEG is well suited to this question in young adult samples because it is non-invasive, relatively scalable, and sensitive to large-scale neural dynamics unfolding on the millisecond timescale. Within resting-state EEG research, microstate analysis identifies brief periods of quasi-stable scalp topography that are commonly interpreted as global functional states of the brain. Importantly, recent cross-study work suggests that the canonical four-class solution is highly reproducible across datasets, supporting the use of conventional microstate classes as a common descriptive framework rather than an idiosyncratic, study-specific representation (22, 23).

To our knowledge, direct comparisons of canonical EEG microstate classes between clinically diagnosed burnout cases and healthy controls remain rare in the burnout literature. Existing EEG studies of burnout have more commonly emphasized spectral power, individual alpha frequency or peak frequency, event-related activity, and connectivity. This gap shaped the present design: we used canonical microstate segmentation not to propose a diagnostic test, but to ask whether burnout severity in a non-clinical higher-education sample is accompanied by measurable variation in whole-brain resting-state temporal dynamics. Microstate analysis is also increasingly relevant to psychiatry. A recent meta-analysis concluded that mood and anxiety disorders show systematic alterations in microstate temporal dynamics, with reduced engagement of class D among the more reproducible findings in mood-disorder samples and altered dynamics in other classes depending on diagnosis and clinical context (24, 25). At the same time, that literature also makes clear that any mapping from microstate classes to specific intrinsic functional networks should be treated as probabilistic rather than literal (23, 26, 27). This caution is especially important in non-clinical student samples, where microstate findings are better interpreted as reflecting altered large-scale brain dynamics than as direct evidence for a single, isolated neural system. Even so, the psychiatric microstate literature provides a strong rationale for testing whether academic burnout, as a stress-related and mental-health-relevant phenotype, shows measurable associations with resting-state microstate dynamics.

This question remains largely unanswered in higher-education research, particularly in Asian university settings that remain underrepresented in the psychobiological literature on academic stress. Although academic burnout has been studied extensively as a psychological and educational outcome, its neurophysiological correlates have rarely been examined in university students, and even less often in designs that model internalizing symptoms and protective resources simultaneously. This gap is important because academic burnout in undergraduates lies at the intersection of educational demands, emerging mental-health burden, and self-regulatory vulnerability. A psychiatry-informed multimodal approach is therefore well suited to this problem because it can situate burnout within the joint study of psychological distress, resilience-related resources, and spontaneous brain dynamics in young adults.

The present study addressed this gap in a multimodal sample of Chinese undergraduates who completed a self-report battery and an eyes-closed resting-state EEG recording. The first aim was to characterize the relationships of academic burnout with trait anxiety, depressive symptoms, and general self-efficacy. The second aim was to determine whether resting-state EEG microstate dynamics were associated with burnout after adjustment for those psychological correlates and demographic covariates. The third aim was to test whether any microstate signal was dimension-general or concentrated in specific components of burnout.

We expected academic burnout to be positively associated with trait anxiety and depressive symptoms and negatively associated with general self-efficacy. We further expected that burnout would show significant associations with microstate temporal parameters after covariate adjustment. Because prior psychiatric microstate work has most consistently implicated classes C and D, we anticipated that any class-specific effects would most likely emerge in that range; however, given the limited prior literature on academic burnout itself, the direction of class-specific effects was treated as tentative rather than strictly confirmatory.

## 2 Methods

### 2.1 Participants and procedure

Participants were undergraduate students recruited from several local colleges in Xinxiang, China. After enrollment, participants completed a self-report battery and subsequently underwent an eyes-closed resting-state EEG recording. For the present report, questionnaire and EEG records were aligned by participant identifier before analysis. Repeated questionnaire records and one repeated EEG acquisition from the same participant were removed during data curation, yielding an ID-aligned multimodal dataset of 335 undergraduates. Because trait anxiety, depressive symptoms, and general self-efficacy contained small amounts of missing data, the primary inferential analyses were conducted on 330 complete cases (94 men, 236 women; mean age = 18.31 years, SD = 0.84).

All participants provided written informed consent prior to participation. The study protocol was approved by the Ethics Committee of Xinxiang Medical University and was conducted in accordance with the Declaration of Helsinki. Testing was conducted individually in a quiet laboratory environment. After the questionnaire assessment, participants were prepared for EEG acquisition and received standardized instructions for the resting-state recording.

### 2.2 Psychological measures

#### 2.2.1 Academic burnout

Academic burnout was assessed with the Learning Burnout of Undergraduates Scale (28), a 20-item instrument developed for Chinese college students. Items are rated on a 5-point Likert scale, with higher scores indicating greater burnout severity. Consistent with the original scale structure, the instrument yields three dimensions—dejection, improper behavior, and low sense of accomplishment—as well as a total score. The total score was designated *a priori* as the primary outcome variable, whereas the three dimension scores were examined in secondary analyses to clarify dimensional specificity.

Because the 20 items are scored from 1 to 5, the possible total score range is 20-100, with higher scores indicating greater academic burnout. According to the original three-dimensional structure, the possible score ranges are 8–40 for dejection, 6–30 for improper behavior, and 6–30 for low sense of accomplishment. In the absence of a diagnostic interview or a universally accepted clinical cutoff for the present sample, burnout scores were primarily analyzed as continuous variables. For descriptive sensitivity analysis only, we additionally classified participants with a total score below 60 as the lower-burnout group and those with a total score of 60 or higher as the higher-burnout group, corresponding to an average item score below versus at or above the scale midpoint.

#### 2.2.2 Trait anxiety

Trait anxiety was measured with the Trait form of the State–Trait Anxiety Inventory (29). The scale contains 20 items rated on a 4-point scale and is intended to index relatively stable individual differences in proneness to anxiety. Higher total scores reflect greater dispositional anxiety.

#### 2.2.3 Depressive symptoms

Depressive symptoms were assessed with the Self-Rating Depression Scale (SDS; 30), a 20-item self-report measure rated on a 4-point scale. Higher total scores indicate more severe depressive symptomatology. To preserve the observed-score metric and avoid introducing additional transformation assumptions into the regression models, the raw total score was used in the primary analyses.

#### 2.2.4 General self-efficacy

General self-efficacy was measured with the General Self-Efficacy Scale (31), which comprises 10 items rated on a 4-point scale. Higher scores indicate a stronger generalized sense of competence in dealing with demands and stressors. When the Discussion refers to perceived coping capacity, it refers specifically to this measured general self-efficacy construct; no broader coping-resource inventory or coping-strategy scale was administered.

All questionnaires were scored according to their original manuals, including reverse scoring where required. In the present study, the psychological block was intentionally restricted to trait anxiety, depressive symptoms, and general self-efficacy, because these variables capture theoretically central vulnerability and resilience dimensions relevant to academic burnout while avoiding unnecessary model proliferation. Internal consistency estimates for all scales in the present sample are reported in the Results section.

### 2.3 EEG acquisition

Resting-state EEG was recorded during an approximately 6-min eyes-closed session using a 64-channel Ag/AgCl cap positioned according to the international 10–20 system. Signals were acquired on a Neuroscan 128-channel system at a sampling rate of 500 Hz, with Cz as the online reference. Electrode impedances were maintained below 5 kΩ. Horizontal and vertical electrooculographic channels were recorded to facilitate the identification and removal of ocular artifacts. Participants were instructed to remain awake, minimize movement, relax their facial muscles, and keep their eyes gently closed throughout the recording.

### 2.4 EEG preprocessing

EEG preprocessing was performed in EEGLAB (v2023.1) running in MATLAB (R2021a), using a standardized resting-state workflow (32). Continuous data were band-pass filtered at 0.5–45 Hz. Grossly contaminated segments were first identified by visual inspection and excluded prior to decomposition. Extended Infomax independent component analysis (runica) was then applied to identify ocular, muscular, and other stereotyped non-neural components on the basis of scalp topography, time course, and spectral characteristics (33). Artifactual components were removed conservatively.

The cleaned data were subsequently re-referenced to the common average reference and segmented into consecutive 2-s epochs. An additional artifact-rejection step was then applied using a ±100 μV threshold, with visual confirmation of rejected epochs. Participants were retained only if at least 4 min of artifact-free resting-state data remained, corresponding to a minimum of 120 epochs. The mean retained artifact-free duration was approximately 5 min. Because the inferential analyses in the present study focused exclusively on broadband microstate dynamics derived from the cleaned sub-50-Hz signal, no frequency-domain findings are reported here.

As a quality-control summary, the number of independent components removed per participant and the amount of retained artifact-free data were inspected before microstate analysis. The retained data length exceeded the predefined minimum of 4 min for all included participants. Participants with insufficient artifact-free data were not retained for the final multimodal analyses. These procedures were used to reduce the likelihood that individual differences in microstate parameters were driven by gross artifacts or unequal data availability.

### 2.5 EEG microstate analysis

Microstate analysis followed standard broadband resting-state procedures derived from the classical segmentation framework (34), the canonical four-class literature (35), and subsequent methodological reviews of EEG microstates (36, 37). Global field power (GFP) peaks were extracted from the preprocessed EEG, and only local GFP maxima were entered into clustering in order to improve topographic signal-to-noise ratio. Adjacent GFP peaks were required to be separated by at least 10 ms to reduce temporal oversampling of highly similar maps.

Microstate clustering was performed using atomize-agglomerate hierarchical clustering (AAHC), first at the single-subject level and then at the grand-average level. All maps were normalized to unit GFP before clustering. A four-class solution was retained *a priori* because it provides the most widely used canonical representation of resting-state EEG microstates and offered an adequate summary of the present data. For descriptive continuity with the conventional literature, the resulting grand-average templates were labeled A–D. This labeling was based on visual correspondence with the canonical four-class microstate framework reported in the resting-state EEG literature. Mean subject-level explained variance for the retained four-class solution was 83.19%.

The continuous EEG was then back-fitted to the grand-average templates, with each time point assigned to the microstate class showing the highest spatial correlation. During back-fitting, polarity was ignored, consistent with standard microstate practice, and segments shorter than 30 ms were reassigned to neighboring segments by temporal smoothing. From the resulting microstate sequence, we derived the conventional class-wise temporal parameters for each microstate class: mean duration, occurrence, and coverage. In the present study, these class-wise temporal parameters were treated as the primary EEG outcomes because they are the most stable, interpretable, and broadly comparable indices across non-clinical and psychiatric microstate studies. Labels A–D were used for descriptive convenience and continuity with the literature, but were not treated as fixed one-to-one proxies for specific functional networks in the statistical analyses. Microstate clustering and back-fitting were implemented in Cartool using the preprocessed EEG data described above, following standard offline resting-state microstate procedures. Duration indexes the average temporal persistence of a given microstate class before transition to another class; occurrence indexes how frequently that class appears per unit time; and coverage indexes the proportion of the recording assigned to that class.

### 2.6 Statistical analysis

All statistical analyses were conducted in Python 3.13.5. Descriptive statistics were computed for demographic variables, questionnaire scores, and EEG microstate features. Scale reliability was evaluated with Cronbach’s alpha. Descriptive data management was conducted on the ID-aligned multimodal dataset of 335 participants. The primary inferential analyses were based on 330 complete cases with non-missing values for academic burnout, demographic covariates, trait anxiety, depressive symptoms, general self-efficacy, and EEG microstate parameters.

The primary inferential objective was to determine whether resting-state EEG microstate dynamics explain variance in academic burnout beyond core psychological correlates. We first examined zero-order associations between academic burnout and candidate psychological variables. On theoretical and empirical grounds, trait anxiety, depressive symptoms, and general self-efficacy were then specified *a priori* as the principal psychological covariates for multimodal analyses.

Next, covariate-adjusted partial correlations were computed between academic burnout and each microstate temporal parameter, controlling for age, sex, trait anxiety, depressive symptoms, and general self-efficacy. To limit false-positive findings due to multiple testing, false discovery rate (FDR) correction was applied across the family of class-wise microstate temporal parameters. Microstate variables showing the clearest and most coherent associations after covariate adjustment were then carried forward into multivariable modeling.

Variable distributions, bivariate scatterplots, residual distributions, variance inflation factors, and influential observations were inspected prior to inferential testing. Multicollinearity was evaluated using variance inflation factors in the final regression model. Because several EEG microstate parameters may show mild distributional deviations, the principal findings were additionally examined using rank-based robustness analyses. The primary regression analyses treated academic burnout total score as a continuous dependent variable. Model 1 included demographic covariates (age and sex). Model 2 added the psychological block (trait anxiety, depressive symptoms, and general self-efficacy). Model 3 added the selected microstate parameters to Model 2 in order to test their incremental explanatory value beyond demographic and psychological factors. Secondary analyses repeated the covariate-adjusted microstate tests for the three academic burnout dimensions (dejection, improper behavior, and low sense of accomplishment) to determine whether the observed EEG associations were dimension-general or dimension-specific.

To address score interpretability and the categorical sensitivity analysis, we conducted a descriptive sensitivity analysis comparing lower- and higher-burnout participants using the LBSU scale-midpoint criterion described above. Group differences in the principal microstate parameters were examined with linear models adjusted for age, sex, trait anxiety, depressive symptoms, and general self-efficacy. These analyses were treated as sensitivity analyses rather than diagnostic case-control analyses because the study did not include clinical interviews or validated clinical burnout cutoffs.

All tests were two-tailed, and statistical significance was set at p <.05, with correction for multiple comparisons where applicable. Given the cross-sectional design, all findings were interpreted as associations and incremental prediction, rather than causal effects.

## 3 Results

### 3.1 Sample characteristics and psychometric properties

The ID-aligned multimodal dataset comprised 335 participants. Because trait anxiety, depressive symptoms, and general self-efficacy contained small amounts of missing data, the primary inferential analyses involving academic burnout, demographic covariates, and EEG microstate parameters were conducted on 330 complete cases. The complete-case sample included 94 men and 236 women, with a mean age of 18.31 years (SD = 0.84). The mean academic burnout total score was 59.62 (SD = 10.94). The corresponding means (SDs) for the three burnout dimensions were 24.14 (5.68) for dejection, 19.00 (3.55) for improper behavior, and 16.48 (3.56) for low sense of accomplishment. The principal psychological covariates showed the expected spread in this student sample: trait anxiety, 45.54 (7.36); depressive symptoms, 37.44 (7.70); and general self-efficacy, 22.82 (4.87).

For score interpretation, the possible LBSU ranges were 20–100 for the total score, 8–40 for dejection, 6–30 for improper behavior, and 6–30 for low sense of accomplishment. Using the scale-midpoint criterion for descriptive purposes, 157 participants (47.6%) were classified as lower-burnout and 173 participants (52.4%) as higher-burnout. This classification was used only to support score interpretation and sensitivity analysis and was not interpreted as a clinical diagnosis.

Internal consistency estimates were acceptable to good for the principal scales. Cronbach’s alpha was 0.873 for academic burnout total, 0.816 for dejection, 0.630 for improper behavior, 0.707 for low sense of accomplishment, 0.840 for trait anxiety, 0.799 for depressive symptoms, and 0.852 for general self-efficacy (**Table 1**). The comparatively lower alpha for improper behavior is consistent with its shorter item set and more heterogeneous behavioral content; notably, this dimension also showed the weakest association with the EEG measures in the subsequent analyses.

**Table 1:** Descriptive statistics, possible score ranges, and internal consistency of the principal study variables in the 330 complete-case sample.

Variable	N	Possible range	Mean	SD	Min	Max	Cronbach’s alpha
Academic burnout total	330	20-100	59.62	10.95	26	92	0.873
Dejection	330	8-40	24.14	5.68	8	38	0.816
Improper behavior	330	6-30	19.00	3.55	9	29	0.630
Low sense of accomplishment	330	6-30	16.48	3.56	7	30	0.707
Trait anxiety	330	20-80	45.54	7.36	26	68	0.840
Depressive symptoms (SDS)	330	20-80	37.44	7.70	20	69	0.799
General self-efficacy	330	10-40	22.82	4.87	11	40	0.852

Descriptive statistics are presented for the 330 complete-case sample used in the primary inferential analyses. Possible range refers to the theoretical score range of each scale or subscale, whereas Min and Max refer to the observed values in the present sample. Cronbach’s alpha values were computed on the same complete-case sample. SDS, Self-Rating Depression Scale.

### 3.2 Zero-order associations among the core psychological variables

Zero-order correlations among academic burnout and the core psychological variables are presented in **Table 2**. Higher academic burnout was associated with higher trait anxiety (r = 0.539, p < 0.001) and higher depressive symptoms (r = 0.526, p < 0.001), and with lower general self-efficacy (r = −0.474, p < 0.001). Trait anxiety and depressive symptoms were themselves positively correlated (r = 0.548, p < 0.001), and both were inversely associated with general self-efficacy (rs = −0.457 and −0.380, respectively; both ps < 0.001). Taken together, these findings indicate that greater academic burnout clustered with greater internalizing distress and with lower general self-efficacy, the measured index of perceived coping capacity in this study.

**Table 2:** Zero-order correlations among academic burnout and the core psychological variables in the 330 complete-case sample.

Variable	Mean	SD	1	2	3	4
1. Academic burnout	59.62	10.95				
2. Trait anxiety	45.54	7.36	0.539			
3. Depressive symptoms (SDS)	37.44	7.70	0.526	0.548		
4. General self-efficacy	22.82	4.87	-0.474	-0.457	-0.380	

Values below the diagonal are Pearson correlations. All correlations shown were significant at p < 0.001.

### 3.3 Covariate-adjusted associations between academic burnout and EEG microstate temporal parameters

**Figure 1:**
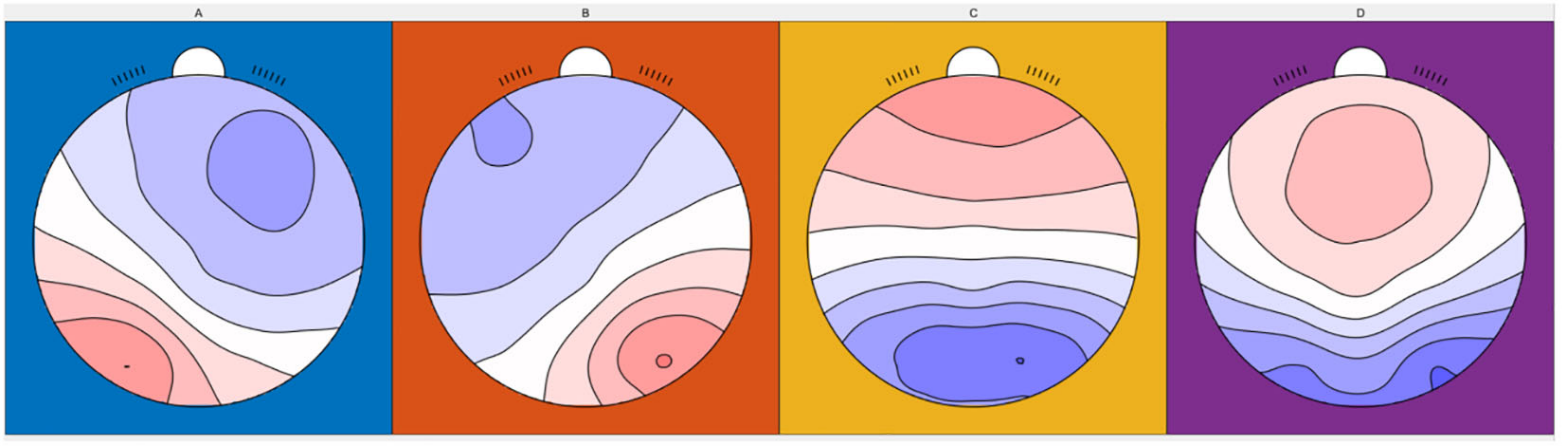
Grand-average canonical EEG microstate maps **(A–D)**. The canonical four-class solution derived from the group-level clustering analysis is shown for descriptive continuity with the resting-state EEG microstate literature. These templates were used for subject-level back-fitting and extraction of temporal parameters. Labels A–D are retained for interpretive clarity and do not imply a fixed one-to-one mapping onto specific functional networks.

**Figure 2:**
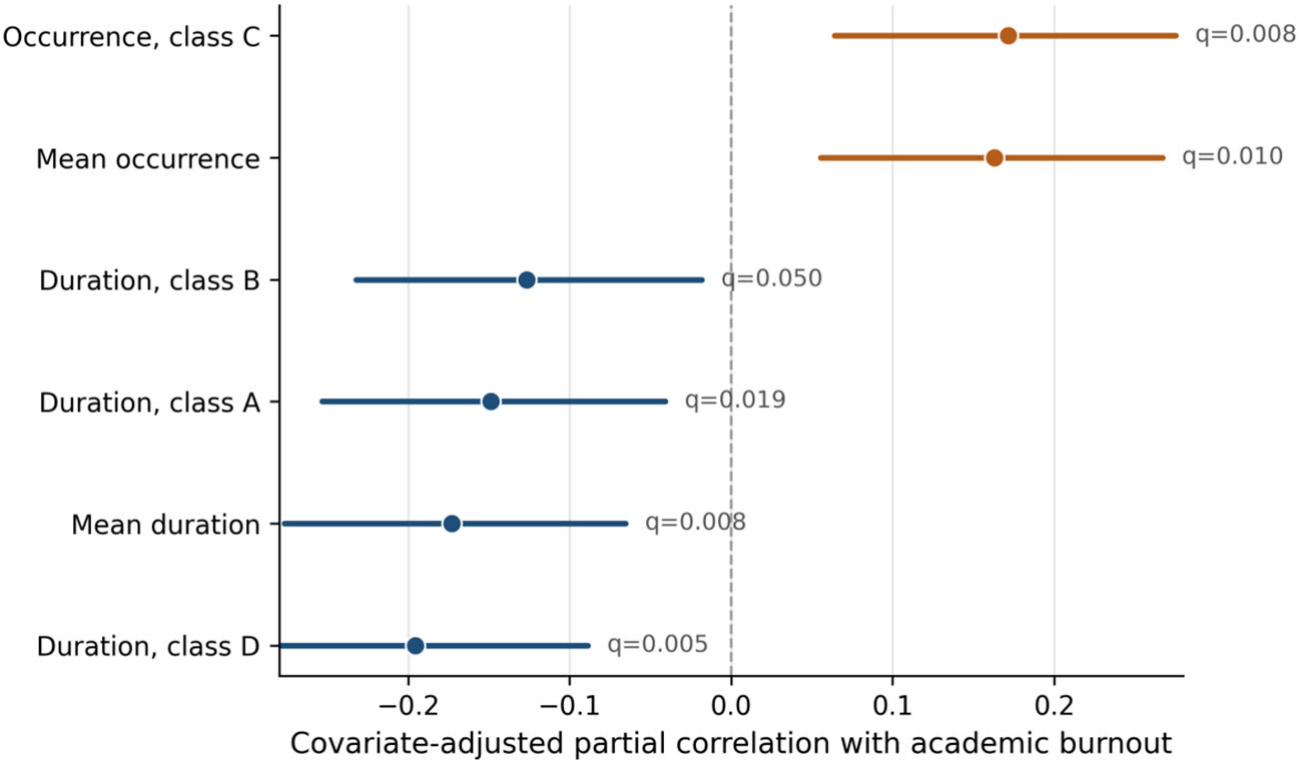
Significant covariate-adjusted microstate correlates of academic burnout. Points denote covariate-adjusted partial correlations between academic burnout total score and the microstate temporal parameters that survived false discovery rate correction. Horizontal lines indicate 95% confidence intervals. Partial correlations were adjusted for age, sex, trait anxiety, depressive symptoms, and general self-efficacy. Negative values indicate that greater burnout was associated with shorter duration, whereas positive values indicate that greater burnout was associated with more frequent occurrence.

The grand-average canonical microstate maps used for back-fitting are shown in **Figure 1**. We next examined whether resting-state EEG microstate dynamics were associated with academic burnout after adjustment for age, sex, trait anxiety, depressive symptoms, and general self-efficacy. The temporal-parameter results relevant to the primary inferential analyses are shown in **Table 3**, and the corrected significant associations are visualized in **Figure 2**.

**Table 3:** Covariate-adjusted associations between academic burnout total score and EEG microstate temporal parameters.

Parameter	N	partial_r	95% CI	p	q
Duration, class D	330	-0.196	[-0.298, -0.089]	<0.001	0.005
Mean duration	330	-0.173	[-0.277, -0.066]	0.002	0.008
Occurrence, class C	330	0.172	[0.064, 0.275]	0.002	0.008
Mean occurrence	330	0.163	[0.055, 0.267]	0.003	0.010
Duration, class A	330	-0.149	[-0.254, -0.041]	0.007	0.019
Duration, class B	330	-0.127	[-0.232, -0.018]	0.021	0.050
Occurrence, class B	330	0.117	[0.009, 0.223]	0.033	0.066
Occurrence, class A	330	0.111	[0.002, 0.217]	0.044	0.077
Coverage, class D	330	-0.090	[-0.197, 0.019]	0.101	0.157
Coverage, class C	330	0.079	[-0.030, 0.186]	0.154	0.216
Duration, class C	330	-0.071	[-0.178, 0.038]	0.198	0.252
Occurrence, class D	330	0.063	[-0.046, 0.171]	0.250	0.292
Coverage, class B	330	0.016	[-0.093, 0.124]	0.778	0.838
Coverage, class A	330	-0.008	[-0.116, 0.101]	0.891	0.891

Partial correlations were adjusted for age, sex, trait anxiety, depressive symptoms, and general self-efficacy. q values are Benjamini–Hochberg false discovery rate adjusted within the temporal-parameter family.

The strongest association was observed for shorter microstate D duration (partial r = −0.196, p < 0.001, q = 0.005). A convergent global pattern was evident, with shorter mean microstate duration also associated with greater burnout severity (partial r = −0.173, p = 0.002, q = 0.008). On the occurrence side, higher academic burnout was associated with more frequent occurrence of microstate C (partial r = 0.172, p = 0.002, q = 0.008) and with higher mean occurrence overall (partial r = 0.163, p = 0.003, q = 0.010). In addition, shorter duration of microstate A (partial r = −0.149, p = 0.007, q = 0.019) and microstate B (partial r = −0.127, p = 0.021, q = 0.050) survived false discovery rate correction. No coverage parameter reached the corrected significance threshold.

To preserve interpretive parsimony in the multivariable stage, the two clearest class-specific signals—microstate D duration and microstate C occurrence—were retained for hierarchical modeling. This decision was guided not only by corrected significance and effect size, but also by their conceptual complementarity as duration-versus-occurrence markers and by their consistency in the follow-up dimensional analyses reported below.

### 3.4 Hierarchical regression predicting academic burnout

**Figure 3:**
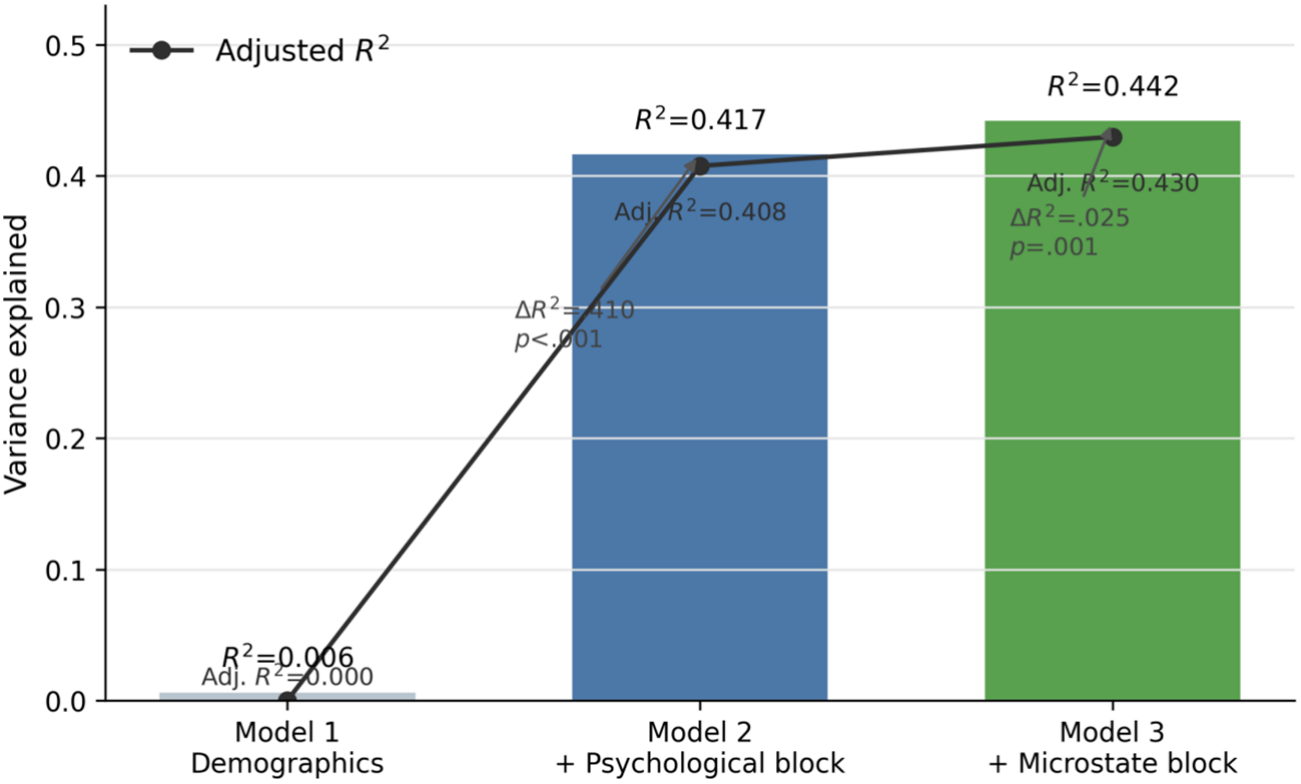
Hierarchical regression models predicting academic burnout. Bars depict the proportion of variance explained (R²) by each model. Model 1 included age and sex; Model 2 additionally included trait anxiety, depressive symptoms, and general self-efficacy; Model 3 further included microstate D duration and microstate C occurrence.

We then tested whether the selected microstate markers explained variance in academic burnout beyond the core psychological variables. Model-comparison results are summarized in **Table 4** and visualized in **Figure 3**. The demographic model (Model 1: age and sex only) explained negligible variance in academic burnout (R² = 0.006, adjusted R² = 0.000, p = 0.359). Adding trait anxiety, depressive symptoms, and general self-efficacy (Model 2) substantially improved model fit (R² = 0.417, adjusted R² = 0.408; ΔR² = 0.410 relative to Model 1, p < 0.001). When microstate D duration and microstate C occurrence were added (Model 3), model fit improved further (R² = 0.442, adjusted R² = 0.430; ΔR² = 0.025 relative to Model 2, p = 0.001).

**Table 4:** Hierarchical regression models predicting academic burnout total score in the 330 complete-case sample.

Panel A. Hierarchical model fit						
Model/Predictor	Specification	R^2^	Adjusted R^2^	ΔR²	Overall model p	p for change
M1	Demographics only	0.006	0.000		0.359	
M2	M1 + trait anxiety + SDS + self-efficacy	0.417	0.408	0.410	<0.001	<0.001
M3	M2 + microstate block (microstate D duration, microstate C occurrence)	0.442	0.430	0.025	<0.001	0.001

Panel B. Final model coefficients (Model 3)						
Predictor	B	SE	β	95% CI	p	VIF
Intercept	35.333	13.621		[8.536, 62.130]	0.010	
Age	0.267	0.556	0.021	[-0.827, 1.361]	0.632	1.05
Sex (male=1)	1.723	1.038	0.071	[-0.319, 3.765]	0.098	1.06
Trait anxiety	0.398	0.079	0.268	[0.243, 0.554]	<0.001	1.64
Depressive symptoms (SDS)	0.418	0.072	0.294	[0.276, 0.560]	<0.001	1.49
General self-efficacy	-0.528	0.110	-0.235	[-0.743, -0.312]	<0.001	1.37
Microstate D duration	-111.631	51.709	-0.111	[-213.363, -9.900]	0.032	1.54
Microstate C occurrence	1.201	0.921	0.067	[-0.610, 3.012]	0.193	1.53

Panel A reports model fit and incremental explained variance; Panel B reports coefficients for the final model (Model 3). VIF values are reported for non-intercept predictors in Model 3. Microstate duration variables in the regression model were entered in seconds; standardized coefficients are provided to aid interpretation.

In the final model, trait anxiety (B = 0.398, β = 0.268, p < 0.001) and depressive symptoms (B = 0.418, β = 0.294, p < 0.001) independently predicted higher academic burnout, whereas general self-efficacy independently predicted lower burnout (B = −0.528, β = −0.235, p < 0.001). Among the EEG variables, shorter microstate D duration remained an independent predictor of greater burnout severity (B = −111.631, β = −0.111, p = 0.032), whereas microstate C occurrence did not retain unique significance once entered simultaneously with the psychological covariates and microstate D duration (B = 1.201, β = 0.067, p = 0.193). Multicollinearity was low (all VIFs ≤ 1.64), indicating that simultaneous inclusion of trait anxiety and depressive symptoms did not compromise model stability.

### 3.5 Dimension-specific analyses of academic burnout

**Figure 4:**
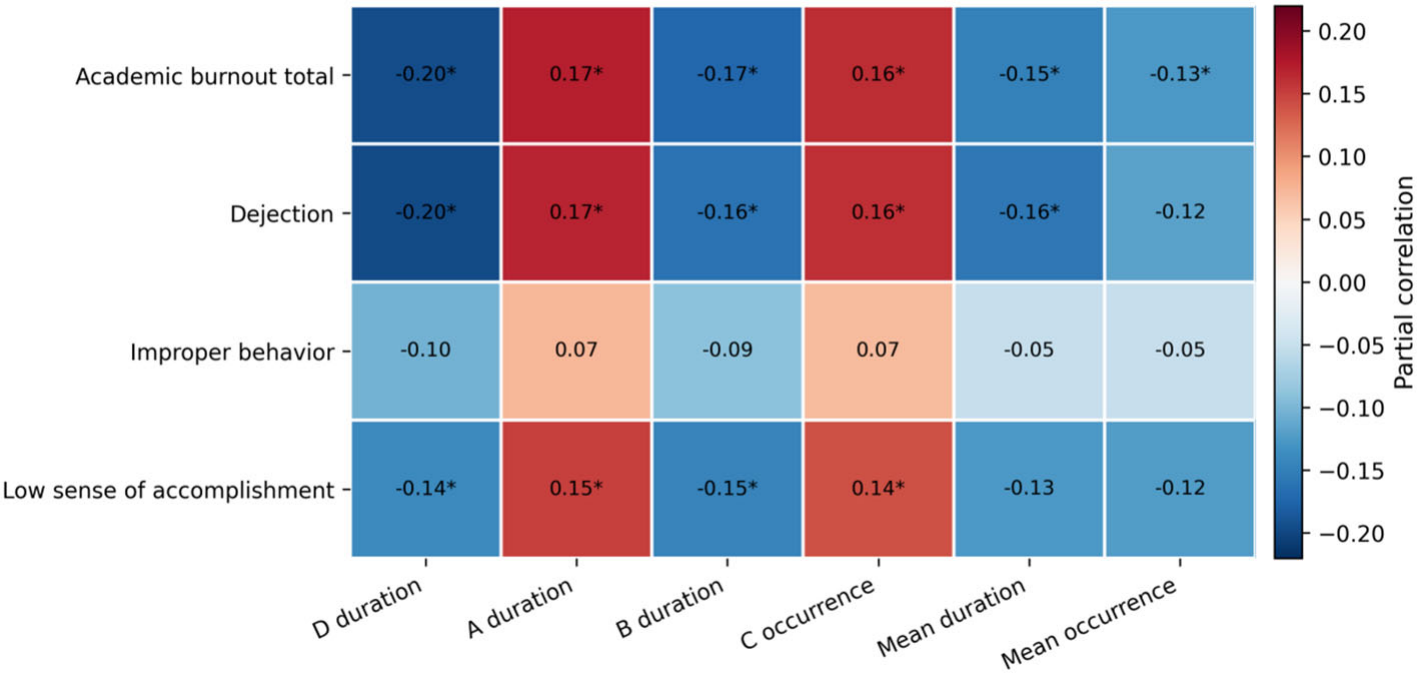
Dimension-specific pattern of covariate-adjusted microstate associations. Cell values represent covariate-adjusted partial correlations for selected microstate temporal parameters across academic burnout total score and its three dimensions. Asterisks indicate false discovery rate-corrected significance within each outcome. All analyses were adjusted for age, sex, trait anxiety, depressive symptoms, and general self-efficacy.

The resulting pattern is summarized in **Table 5** and visualized in **Figure 4**. Panel A of **Table 5** presents the dimension-specific covariate-adjusted associations, whereas Panel B summarizes the rank-based robustness analyses for the principal findings.

**Table 5:** Dimension-specific covariate-adjusted associations for selected EEG microstate parameters and rank-based robustness analyses.

Panel A. Dimension-specific covariate-adjusted associations				
Outcome	Parameter	partial_r	p	q
Academic burnout total	Duration, class D	-0.196	<0.001	0.005
Academic burnout total	Occurrence, class C	0.172	0.002	0.008
Academic burnout total	Mean duration	-0.173	0.002	0.008
Academic burnout total	Mean occurrence	0.163	0.003	0.010
Academic burnout total	Duration, class A	-0.149	0.007	0.019
Academic burnout total	Duration, class B	-0.127	0.021	0.050
Dejection	Duration, class D	-0.197	<0.001	0.004
Dejection	Occurrence, class C	0.167	0.002	0.012
Dejection	Mean duration	-0.162	0.003	0.012
Dejection	Mean occurrence	0.161	0.003	0.012
Dejection	Duration, class A	-0.156	0.004	0.012
Dejection	Duration, class B	-0.118	0.031	0.063
Improper behavior	Duration, class D	-0.104	0.059	0.644
Improper behavior	Occurrence, class C	0.073	0.187	0.644
Improper behavior	Mean duration	-0.090	0.102	0.644
Improper behavior	Mean occurrence	0.069	0.210	0.644
Improper behavior	Duration, class A	-0.050	0.365	0.644
Improper behavior	Duration, class B	-0.051	0.357	0.644
Low sense of accomplishment	Duration, class D	-0.140	0.011	0.038
Low sense of accomplishment	Occurrence, class C	0.151	0.006	0.038
Low sense of accomplishment	Mean duration	-0.145	0.008	0.038
Low sense of accomplishment	Mean occurrence	0.140	0.011	0.038
Low sense of accomplishment	Duration, class A	-0.127	0.021	0.059
Low sense of accomplishment	Duration, class B	-0.123	0.026	0.060

Panel B. Rank-based robustness analyses for academic burnout total score				
Outcome	Microstate parameter	Spearman’s ρ	p	
Academic burnout total	Duration, class D	-0.113	0.040	
	Occurrence, class C	0.131	0.017	
	Mean duration	-0.114	0.039	
	Mean occurrence	0.110	0.045	
	Duration, class A	-0.126	0.022	

Panel A presents covariate-adjusted partial correlations between academic burnout outcomes and selected EEG microstate temporal parameters. Partial correlations were adjusted for age, sex, trait anxiety, depressive symptoms, and general self-efficacy. False discovery rate (FDR) correction was applied separately within each outcome across the family of tested microstate temporal parameters. Panel B presents rank-based robustness analyses for the principal findings using Spearman’s rho for the academic burnout total score. All analyses were based on the 330 complete-case sample. FDR-adjusted q values are reported to facilitate identification of statistically robust associations.

The clearest pattern emerged for dejection, the affective core of academic burnout. Higher dejection was associated with shorter microstate D duration (partial r = −0.197, p < 0.001, q = 0.004), higher microstate C occurrence (partial r = 0.167, p = 0.002, q = 0.012), shorter mean duration (partial r = −0.162, p = 0.003, q = 0.012), higher mean occurrence (partial r = 0.161, p = 0.003, q = 0.012), and shorter microstate A duration (partial r = −0.156, p = 0.004, q = 0.012). A second, somewhat weaker but still reliable pattern was observed for low sense of accomplishment. Higher scores on this dimension were associated with higher microstate C occurrence (partial r = 0.151, p = 0.006, q = 0.038), shorter mean duration (partial r = −0.145, p = 0.008, q = 0.038), higher mean occurrence (partial r = 0.140, p = 0.011, q = 0.038), and shorter microstate D duration (partial r = −0.140, p = 0.011, q = 0.038). By contrast, no microstate temporal parameter survived correction for improper behavior.

Taken together, these dimension-specific analyses indicate that the covariate-adjusted microstate associations were not evenly distributed across all LBSU dimensions. They were observed mainly for dejection and low sense of accomplishment, whereas improper behavior showed no corrected association. In the LBSU framework, improper behavior refers to maladaptive study-related behaviors and behavioral disengagement, not to moral misconduct. Thus, the EEG findings appear to map more strongly onto the affective-exhaustive and self-evaluative components of burnout than onto its behavioral-disengagement component.

### 3.6 Robustness analysis

The principal microstate findings remained directionally consistent in rank-based analyses (**Table 5B**). Higher academic burnout was associated with shorter microstate D duration (Spearman’s ρ = −0.113, p = 0.040) and higher microstate C occurrence (ρ = 0.131, p = 0.017). Mean duration (ρ = −0.114, p = 0.039), mean occurrence (ρ = 0.110, p = 0.045), and microstate A duration (ρ = −0.126, p = 0.022) showed the same directional pattern. Although these nonparametric effect sizes were modest, their consistency with the primary covariate-adjusted analyses supports the robustness of the overall temporal-dynamic signal.

### 3.7 Descriptive lower- versus higher-burnout sensitivity analysis

**Figure 5:**
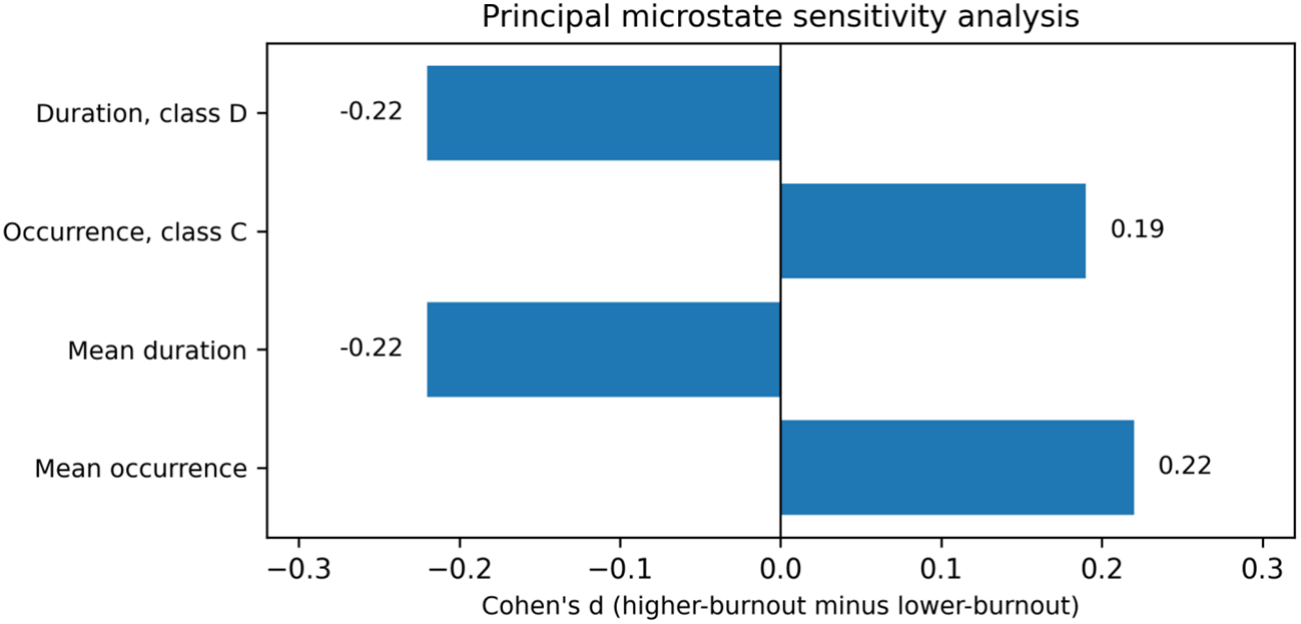
Standardized lower- versus higher-burnout group differences in the principal microstate sensitivity analysis. Values represent Cohen’s d for higher-burnout minus lower-burnout groups. Negative values indicate shorter duration in the higher-burnout group; positive values indicate higher occurrence in the higher-burnout group.

We next conducted a descriptive sensitivity analysis to address the distribution of lower- and higher-burnout participants while avoiding clinical labels not supported by the study design. Participants with LBSU total scores below 60 were classified as lower-burnout (n = 157, 47.6%), and those with scores of 60 or higher were classified as higher-burnout (n = 173, 52.4%). Compared with the lower-burnout group, the higher-burnout group showed shorter class D duration (66.18 ± 9.85 ms vs. 68.54 ± 11.89 ms; Cohen’s d = -0.22). In a covariate-adjusted linear model controlling for age, sex, trait anxiety, depressive symptoms, and general self-efficacy, the higher-burnout group showed shorter class D duration (adjusted B = -3.38 ms, 95% CI [-6.12, -0.64], p = 0.016, q = 0.028). Class C occurrence was descriptively higher in the higher-burnout group (3.95 ± 0.63 vs. 3.84 ± 0.59 per second; Cohen’s d = 0.19), but the adjusted contrast did not reach conventional significance (adjusted B = 0.137 per second, 95% CI [-0.017, 0.292], p = 0.082, q = 0.082). Mean duration and mean occurrence showed the same directionally consistent pattern (**Table 6**, **Figure 5**). When the full exploratory set of 14 temporal parameters was considered, the strongest group contrasts remained nominally significant but did not reach q < 0.05 after FDR correction (minimum q = 0.074); therefore, the group-based results are interpreted as supportive rather than definitive.

**Table 6:** Descriptive lower- versus higher-burnout sensitivity analysis for principal EEG microstate parameters.

Parameter	Lower-burnout M ± SD	Higher-burnout M ± SD	Cohen’s d	Adjusted difference (95% CI); p; q
Duration, class D	68.54 ± 11.89 ms	66.18 ± 9.85 ms	-0.22	-3.38 ms [-6.12, -0.64]; p = 0.016; q = 0.028
Occurrence, class C	3.84 ± 0.59/s	3.95 ± 0.63/s	0.19	0.137/s [-0.017, 0.292]; p = 0.082; q = 0.082
Mean duration	70.68 ± 8.77 ms	68.77 ± 8.81 ms	-0.22	-2.64 ms [-4.86, -0.43]; p = 0.020; q = 0.028
Mean occurrence	14.76 ± 1.73/s	15.16 ± 1.83/s	0.22	0.528/s [0.080, 0.977]; p = 0.021; q = 0.028

Lower-burnout was defined as LBSU total score < 60 and higher-burnout as LBSU total score ≥ 60. Adjusted differences were estimated as higher-burnout minus lower-burnout from linear models controlling for age, sex, trait anxiety, depressive symptoms, and general self-efficacy. q values are FDR-adjusted across the four principal sensitivity tests. This grouping was descriptive and should not be interpreted as a clinical diagnosis.

### 3.8 Summary of the main findings

In summary, academic burnout in this higher-education sample was strongly related to established psychological vulnerability and resilience factors, particularly trait anxiety, depressive symptoms, and general self-efficacy. Beyond these psychological correlates, resting-state EEG microstate dynamics provided additional explanatory value. The most consistent neural signal was reduced microstate D duration, accompanied by a broader temporal pattern of shorter mean microstate duration and more frequent occurrence, with class C occurrence representing the clearest class-specific occurrence marker. Dimension-specific analyses further indicated that these EEG correlates were concentrated in the dejection and low-accomplishment components of burnout. The lower- versus higher-burnout sensitivity analysis was directionally consistent with the continuous findings, particularly for class D duration, but it should be interpreted as a descriptive analysis rather than as a diagnostic case-control comparison. This pattern supports a psychobiologically informed account of academic burnout in higher education.

## 4 Discussion

### 4.1 Principal findings

In this multimodal sample of 330 Chinese undergraduates, academic burnout was primarily associated with internalizing distress and lower general self-efficacy, and resting-state EEG microstate dynamics added only a small but statistically reliable increment beyond those psychological correlates. The principal EEG finding was shorter class D duration. In practical terms, this means that the class-D scalp configuration persisted for fewer milliseconds on average before transitioning to another microstate. Its association with burnout remained after adjustment for age, sex, trait anxiety, depressive symptoms, and general self-efficacy, suggesting that students with comparable levels of internalizing distress and perceived coping capacity still showed slight burnout-related differences in resting-state temporal dynamics. The dimension-specific results further clarify this interpretation: the adjusted microstate associations were observed mainly for dejection and low sense of accomplishment, not for improper behavior. By an integrative view, we therefore mean that burnout in this sample was characterized at three connected measured levels: psychological vulnerability indexed by trait anxiety and depressive symptoms, perceived coping capacity indexed by general self-efficacy, and a modest resting-state temporal-dynamic EEG correlate. This does not mean that the present cross-sectional data identify a causal mechanism, an institutional pathway, or a diagnostic EEG signature; rather, they indicate that academic burnout can be studied as a stress-related higher-education phenotype linking self-reported distress, self-evaluative capacity, and large-scale spontaneous brain-state dynamics.

### 4.2 Academic burnout as a mental-health-relevant condition in higher education

The psychological findings are consistent with higher-education mental-health research showing that academic burnout is closely linked to anxiety, depressive symptoms, and self-evaluative resources such as self-efficacy in Chinese undergraduate samples (18–20). Longitudinal evidence also suggests that academic stress prospectively contributes to burnout over time (38). In our data, trait anxiety, depressive symptoms, and lower general self-efficacy accounted for a substantial proportion of the variance in burnout. This pattern indicates that burnout in university students is not simply an academic-performance construct; it is closely embedded in the broader internalizing mental-health burden of higher education.

The role of general self-efficacy is also important for interpreting the term perceived coping capacity. We did not measure coping resources broadly, such as problem-focused coping, emotion-focused coping, social support, or institutional support. Rather, general self-efficacy was used as a focused self-report index of students’ perceived ability to handle demands and stressors. The negative association between self-efficacy and burnout therefore suggests that students who felt less capable of managing demands tended to report greater burnout, even after anxiety and depressive symptoms were considered.

At the same time, burnout should not be conceptualized in purely intrapsychic terms. Academic burnout emerges within institutional environments that shape workload, evaluation pressure, access to support, and opportunities for recovery. The present study did not directly measure these institutional variables, and this is an important limitation. Accordingly, our integrative account should be understood as an individual-level psychobiological contribution to a broader multilevel framework, not as a complete institutional model. Future studies should combine psychobiological measures such as EEG microstates with direct measures of educational context, including examination periods, workload intensity, perceived institutional support, academic support, recovery opportunities, and institutional mental-health resources.

### 4.3 Interpretation of the microstate findings

The principal EEG temporal-dynamic finding was shorter microstate D duration. A microstate duration result is a temporal-dynamic result: it means that the corresponding scalp-potential configuration remained stable for a shorter average period before the EEG transitioned to another configuration. Thus, shorter class D duration should be interpreted as reduced temporal persistence of a canonical resting-state configuration, not as evidence that a single brain region or network is impaired. The finding remained significant after adjustment for age, sex, trait anxiety, depressive symptoms, and general self-efficacy and retained independent significance in the final model. In practical terms, this means that burnout severity was associated with a small amount of class-D temporal instability that was not fully explained by the measured psychological variables. The effect was modest, as shown by the standardized coefficient for class D duration (β = −0.111) and the 2.5% increase in explained variance after adding the microstate block. Higher microstate C occurrence was a reliable covariate-adjusted correlate in the continuous analysis, but it did not remain independently significant in the final regression model and did not reach conventional significance in the adjusted group contrast. The most defensible interpretation is therefore selective and restrained: class D duration was the most stable EEG correlate, whereas class C occurrence was a secondary, less independent temporal feature.

Recent syntheses of the clinical and psychiatry-related microstate literature provide a useful, but necessarily cautious, context for these findings. A meta-analysis of mood and anxiety disorders reported systematic microstate alterations across internalizing conditions (24), and a broader 2025 systematic review and meta-analysis concluded that classes C and D are among the most repeatedly altered microstates across psychiatric spectra, while also emphasizing heterogeneity across diagnoses and states (25). At the same time, recent reviews of the functional microstate literature have stressed that class-to-network mappings are probabilistic rather than definitive and should not be treated as direct substitutes for source-localized network measures (23, 39). This caution is especially important in a non-clinical student sample such as ours.

One cautious functional interpretation is that the class D result may relate to reduced temporal stability of control-related resting-state configurations. Microstate D has often been linked, in a broad and non-exclusive sense, to top-down attentional or executive-control processes, whereas microstate C has more often been discussed in relation to salience-related, interoceptive, or self-referential processing (26, 27). Because these class-to-network mappings are probabilistic rather than definitive, the present data cannot demonstrate a specific neural mechanism of burnout. Instead, they support a narrower conclusion: students with higher burnout showed small but statistically reliable differences in the temporal organization of spontaneous EEG microstates, especially reduced persistence of class D. These differences should be treated as adjunctive psychobiological correlates of burnout severity, not as a disorder-specific mechanism, diagnostic EEG signature, or clinical classifier.

### 4.4 Why microstate associations were most evident in dejection and low accomplishment

The dimensional analyses sharpened the interpretation of the main effect by showing where the microstate associations were located within the burnout construct. The clearest associations were observed for dejection, followed by low sense of accomplishment, whereas improper behavior showed no corrected association with microstate temporal parameters. This pattern is psychologically coherent. Dejection reflects emotional exhaustion, low mood, and subjective depletion, and therefore lies closest to the internalizing spectrum indexed here by trait anxiety and depressive symptoms. Low sense of accomplishment reflects self-evaluative failure and diminished efficacy beliefs, which explains why it is conceptually close to general self-efficacy. Improper behavior, in contrast, refers to behavioral disengagement and maladaptive study-related behaviors. This component may depend more strongly on study habits, classroom demands, social regulation, and situational opportunity than on resting-state temporal brain dynamics alone.

This dimensional pattern strengthens the interpretability and mental-health relevance of the findings. If the microstate associations had been equally strong across all burnout dimensions, they would be harder to distinguish from a nonspecific correlate of general symptom reporting. Instead, the associations were concentrated in the affective-exhaustive and self-evaluative components of burnout. In concrete terms, this means that the EEG findings aligned more closely with feeling emotionally depleted and ineffective than with overt behavioral disengagement. The results therefore support a heterogeneous view of academic burnout in which its most psychiatry-relevant features are concentrated in affective and self-evaluative dimensions, while its behavioral component may require additional contextual and behavioral measures.

### 4.5 Clinical and educational implications

The present findings have implications for both research and student mental-health practice, but these implications should be kept proportionate to the modest effect sizes. Resting-state microstates should not be used as stand-alone screening or diagnostic tools for academic burnout. Their potential value is adjunctive: they may help future studies characterize how burnout relates to spontaneous brain-state dynamics when combined with psychological symptoms, self-efficacy, sleep and recovery, academic workload, perceived institutional support, and contextual measures of academic pressure.

Second, the concentration of the microstate associations in dejection and low sense of accomplishment highlights the importance of addressing the affective and self-evaluative core of burnout rather than focusing only on observable study behavior. Third, because burnout is shaped by educational context as well as individual vulnerability, preventive efforts in higher education should combine student-level support with institution-level strategies such as manageable workload design, timely access to mental-health support, academic feedback practices that support efficacy, and recovery-promoting academic environments. In this sense, the present results support a psychiatry-informed but context-sensitive approach to academic stress in university settings.

### 4.6 Strengths, limitations, and future directions

Several features of the study strengthen confidence in the findings. The multimodal sample is comparatively large for a resting-state EEG microstate study in student mental health. Questionnaire and EEG records were aligned at the participant level, duplicate records were removed, and the primary models were re-estimated on a strict complete-case sample, yielding results that were essentially unchanged from the broader aligned sample. EEG acquisition and preprocessing followed a standardized workflow, and the inferential strategy was deliberately restrained: we focused on canonical four-class resting-state microstates, controlled the main analyses for theoretically central psychological covariates, and applied false discovery rate correction to reduce the risk of false-positive interpretation.

The study also has important limitations. First, the cross-sectional design precludes causal inference. We cannot determine whether altered microstate dynamics increase vulnerability to burnout, reflect the effects of sustained academic distress, or both. Second, participants were recruited from colleges in one Chinese city, and the sample was female-skewed; generalizability to other institutional, regional, and cultural contexts remains uncertain. Third, the psychological assessment relied on self-report measures only. We did not have clinician-rated psychopathology, structured diagnostic interviews, objective academic performance indicators, or repeated measurements across academic stress cycles. Fourth, although the main covariates were theoretically justified and statistically well behaved, several potentially relevant influences were not measured, including sleep quality, recent examination load, caffeine use, menstrual-cycle effects, socioeconomic conditions, and concurrent life stress. These factors may plausibly influence both burnout severity and resting-state EEG dynamics, and therefore the present associations should be interpreted as preliminary psychobiological correlates rather than condition-specific signatures. Fifth, although we interpreted general self-efficacy as an index of perceived coping capacity, the study did not measure coping strategies, social support, institutional support, or other coping resources directly; future work should include these variables to test a fuller coping-resource model. Finally, because the analyses intentionally centered on conventional class-wise temporal parameters, the present study does not address source-level mechanisms, higher-order syntax, or other dynamic features that may also prove informative.

Moreover, the lower- versus higher-burnout categorization was based on the LBSU scale midpoint and was introduced only to improve score interpretability and respond to the need for a categorical sensitivity analysis. Because no structured clinical interview was conducted and no universally accepted clinical cutoff was available for this sample, these categories should not be interpreted as healthy controls versus clinical burnout cases.

These limitations also indicate the most informative next steps. Longitudinal studies spanning routine teaching periods, examination windows, and recovery phases are needed to test whether microstate alterations track state-related fluctuations in academic stress and burnout. Future work should integrate sleep, recent workload, life stress, and institution-level variables, and should examine whether psychobiological measures add practical value in intervention studies or mixed-methods designs. Replication across universities, disciplines, and cultural settings—especially across underrepresented higher-education contexts in Asia and beyond—will be necessary before the present pattern can be regarded as a robust correlate of academic burnout.

Artificial intelligence and machine-learning methods may also be useful in future multimodal research on academic burnout. Rather than examining each EEG parameter in isolation, supervised models could combine microstate temporal features with psychological symptoms, self-efficacy, sleep, workload, and institutional-context variables to estimate individualized burnout risk or recovery trajectories. However, such applications require sufficiently large and preferably multi-site samples, nested cross-validation, external validation, calibration assessment, transparent feature selection, and interpretable reporting. Because the present study was cross-sectional and did not include diagnostic labels, we did not train classifiers in the current report; longitudinal studies are needed to test whether multimodal machine-learning models improve prediction beyond self-report measures alone while avoiding overfitting and premature clinical claims.

## 5 Conclusion

In conclusion, academic burnout in this sample of Chinese undergraduates was strongly associated with trait anxiety, depressive symptoms, and lower general self-efficacy, but it was not fully captured by those psychological correlates. Resting-state EEG microstate dynamics added a small but statistically reliable increment, with the most stable finding being shorter class D duration. This finding means that higher burnout was associated with reduced average temporal persistence of the class-D resting-state configuration after adjustment for key psychological variables. The microstate associations were most evident for dejection and low sense of accomplishment, suggesting closer alignment with the affective-exhaustive and self-evaluative aspects of burnout than with behavioral disengagement. These findings support a cautious integrative account of academic burnout as a higher-education stress phenotype involving internalizing distress, reduced perceived coping capacity, and modest psychobiological variation. They do not support a disorder-specific EEG mechanism, diagnostic EEG signature, or clinical EEG classifier, but they suggest that multimodal approaches may enrich future longitudinal and intervention-oriented research on academic stress in university settings.

## Data Availability

The raw data supporting the conclusions of this article will be made available by the authors, without undue reservation.
